# Pulmonary Arterial Hypertension Associated With Chronic Mephedrone Use: A Case Report

**DOI:** 10.1002/pul2.70415

**Published:** 2026-10-01

**Authors:** Masoud Kashoub, Michael Plunkett, Heba Nashat, Konstantinos Dimopoulos, Colm McCabe, Carl Harries, S. John Wort, Paul I. Dargan, Laura C. Price

**Affiliations:** ^1^ Clinical Fellow Respiratory Medicine, Royal Brompton Hospital Part of Guy's and St Thomas' NHS Foundation Trust London London UK; ^2^ National Pulmonary Hypertension Service, Royal Brompton Hospital Part of Guy's and St Thomas' NHS Foundation Trust London UK; ^3^ National Heart and Lung Institute, Guy Scadding Building Imperial College London London London UK; ^4^ Clinical Toxicology Guy's and St Thomas' NHS Foundation Trust London UK; ^5^ Faculty of Life Sciences and Medicine King's College London London UK

**Keywords:** drug‐induced PAH, Methamphetamines, PAH

## Abstract

Mephedrone is a synthetic cathinone with sympathomimetic effects, but has not previously been linked to pulmonary arterial hypertension (PAH). We report a 42‐year‐old woman with no prior cardiopulmonary disease who presented with severe pre‐capillary PAH after a decade of near‐daily mephedrone use. Extensive evaluation—including autoimmune, infectious, genetic, and imaging studies—excluded recognised causes of PAH. Following cessation of mephedrone and initiation of dual oral PAH‐targeted therapy, she demonstrated substantial initial clinical and haemodynamic improvement; however, the response remained incomplete, necessitating further treatment escalation with selexipag. This appears to be the first reported case of PAH linked to mephedrone use.

## 1 Introduction

Mephedrone (4‐methylmethcathinone) is a synthetic cathinone with sympathomimetic activity that has structural and pharmacological similarities to amphetamines [1, 2, 3]. Its acute cardiovascular effects (tachycardia, hypertension, chest discomfort, and arrhythmias) are well documented in laboratory studies, controlled human exposures, and acute toxicity presentations [3, 4, 5, 6, 7, 8, 9]. Despite these cardiovascular effects, no previous reports have identified pulmonary hypertension associated with mephedrone use [1, 2, 3, 4, 5, 6, 7, 8, 9]. In contrast, stimulants such as amphetamines and anorexigens (e.g., fenfluramine) are recognised as potential causes of drug‐induced PAH [10, 11], producing progressive vascular remodelling and right‐heart failure [12, 13].

We report a case of pulmonary hypertension associated with chronic mephedrone use that, despite initial clinical and hemodynamic improvement following drug cessation and PAH‐targeted therapy, remained irreversible, with persistent pulmonary vascular disease requiring further therapeutic escalation.

## 2 Case Presentation

A 42‐year‐old woman, a school teacher, was referred to our pulmonary hypertension service; she had been previously healthy with no history of cardiopulmonary disease. Her shortness of breath began suddenly after a viral‐like illness, accompanied by mild peripheral oedema which had progressed over several weeks. At her first specialist‐centre assessment, she was in WHO functional class III. Her history revealed a decade‐long pattern of near‐daily mephedrone use, typically three to four lines by nasal insufflation on most evenings. This had been her primary recreational substance, with only occasional cocaine exposure. She had stopped using mephedrone approximately 5 weeks prior to review and had also recently stopped smoking. She previously consumed around 15 cigarettes per day. Additionally, she had reduced her alcohol consumption to 4 days per week, averaging approximately 12 units weekly.

There was no family history of pulmonary hypertension or connective tissue disease, and no exposure to anorexigens, chemotherapeutic agents, or occupational toxins. There was no history of travel prior to her illness.

Examination showed an elevated jugular venous pressure and loud P2 but no peripheral oedema or connective tissue disease stigmata. Her 6‐min walk test (6MWT) was 445 m, desaturating from 97% to 91% on room air. Laboratory testing for liver function, autoimmune serology, viral screening including HIV, and a full PAH genetic panel (Table 1) were negative. Liver ultrasound showed no features suggestive of cirrhosis. Initial CTPA at the referring centre showed CT features of right heart strain and a dilated main pulmonary artery, measuring 36 mm. Initial echocardiography demonstrated a markedly dilated and dysfunctional right ventricle with severe tricuspid regurgitation. Repeat echocardiography and cardiac MRI confirmed severe right heart dilation, right ventricular dysfunction, and features of pulmonary hypertension (Table 2).

**TABLE 1:** Laboratory, microbiology, immunology, and functional parameters at diagnosis and follow‐up.

Parameter	At time of diagnosis	3 months follow‐up	6 months follow‐up	12 months follow‐up
Haemoglobin (g/L)	138	—	—	
Platelet count (×10^9^/L)	148			
INR (ratio)	1			
Albumin (g/L)	45			
BNP (pg/mL)	309	240	—	
NT‐proBNP (pg/mL)	—	1373	373	91
WHO Functional Class	III	II	II	I
6‐min walk distance (m)	445 m	—	555 m	654 m
ANA	Negative	—	—	
ENA panel	All negative	—	—	
Rheumatoid factor	< 20	—	—	
U1RNP	1.8 (normal)	—	—	
Anti‐RNP70	2.7 (normal)	—	—	
HIV Ag/Ab	Negative	Negative	—	
Hepatitis B surface Ag	Negative	—	—	
Hepatitis B core Ab	Negative	—	—	
Hepatitis C Ab	Negative	—	—	
Microbiology	No infectious cause identified	—	—	
Genetic testing for PAH	—	—	Negative	

*Note:* Table 1 This table summarises the patient's baseline haematologic values, cardiac biomarkers, immunologic and microbiologic screening, and functional capacity at the time of diagnosis (March 2025), with interval assessments in June and October 2025.

Abbreviations: ANA, antinuclear antibody; Anti‐RNP70, anti‐ribonucleoprotein 70 antibody; BNP, B‐type natriuretic peptide; ENA, extractable nuclear antigen; Hepatitis B core Ab, hepatitis B core antibody; Hepatitis B surface Ag, hepatitis B surface antigen; Hepatitis C Ab, hepatitis C antibody; HIV Ag/Ab, human immunodeficiency virus antigen/antibody combination assay; INR, international normalised ratio; NT‐proBNP, N‐terminal pro‐B‐type natriuretic peptide; PAH, pulmonary arterial hypertension; U1RNP, U1 ribonucleoprotein antibody; WHO FC, World Health Organization Functional Class.

**TABLE 2:** Integrated diagnostic and follow‐up parameters.

Modality	Parameter	At time of diagnosis	Follow‐up	16 months follow‐up
ECHO	Right atrial area (cm^2^)	29	20.4	
	Right ventricular end‐diastolic diameter (cm)	5.20 (severely dilated)	3.80 (moderately dilated)	
	RV function	Impaired	Improved	
	Septal morphology	Marked septal flattening, d‐shaped LV	Partial normalisation	
	TR Vmax (m/s)	4.8	4.21	
	Estimated PASP (mmHg)	99	74	
CMR	RA indexed area (cm^2^/m^2^)	18	—	
	RV ejection fraction	36%	—	
	LV ejection fraction	64%	—	
	Main pulmonary artery diameter (mm)	30	—	
PFT	FEV_1_ L (% predicted)	2.95 (92.5)	—	
	FVC L (%)	3.82 (97.1)	—	
	FEV_1_/FVC (%)	77%	—	
	TLC L (%)	5.61 (99.2)	—	
	DLCO (%)	105% predicted	—	
RHC	RA pressure (mmHg)	10	4	6
	Mean pulmonary artery pressure (mmHg)	55	37	39
	PCWP (mmHg)	3	7	10
	Cardiac index (Thermodilution) (L/min/m^2^)	1.89	2.6	3.5
	PVR (Wood units)	11.5 WU	~5.7 WU	4 WU

*Note:* Table 2 This table summarises the patient's multimodality cardiopulmonary evaluation at diagnosis (March 2025) and follow‐up (October 2025), incorporating echocardiography, cardiac MRI, pulmonary function testing, and invasive hemodynamics.

Abbreviations: CI, cardiac index; CMR, cardiac magnetic resonance; DLCO, diffusing capacity for carbon monoxide; FEV1, forced expiratory volume in 1 second; FVC, forced vital capacity; LV, left ventricle; PAH, pulmonary arterial hypertension; PASP, pulmonary artery systolic pressure; PCWP, pulmonary capillary wedge pressure; PFT, pulmonary function test; PVR, pulmonary vascular resistance; RA, right atrium; RHC, right heart catheterisation; RV, right ventricle; TLC, total lung capacity.

Right heart catheterisation confirmed severe pre‐capillary pulmonary arterial hypertension with markedly elevated pulmonary vascular resistance, reduced cardiac index, and no vasoreactivity to inhaled nitric oxide. Pulmonary function tests were within normal range, including gas transfer, and ventilation–perfusion SPECT–CT excluded chronic thromboembolic disease. Relevant haemodynamic findings and parameters are summarised in Table 2.

She was commenced on dual oral upfront sequential PAH therapy, tadalafil (40 mg daily) and macitentan (10 mg daily), and diuretics. Over subsequent months, she reported symptomatic improvement. Her 6MWT increased to 555 m, with oxygen saturation improving from 99% at baseline to 100% after the test, and natriuretic peptide levels declined (Table 1). Her 5 months follow‐up echocardiography showed improvement in right ventricular size and tricuspid regurgitation. Repeat right heart catheterisation 7 months later demonstrated substantial haemodynamic improvement, including reductions in mean pulmonary artery pressure and pulmonary vascular resistance with an increased cardiac index (Table 2). Due to residual intermediate low‐risk PAH, selexipag was initiated at 200 mcg twice daily, with PH nurse‐led titration increasing the dose to 800 mcg twice daily. She remained abstinent from mephedrone.

After 3 months of triple therapy, she demonstrated a marked clinical response, improving to WHO functional class I, with an increase in 6MWT to 654 m and a reduction in NT‐proBNP to 91 pg/mL (Table 1). Given the favourable clinical and biochemical response, repeat RHC was performed several months later to assess her hemodynamic response and guide potential treatment de‐escalation. Despite modest improvement in PVR, RHC demonstrated persistent pulmonary vascular disease (Table 2). Therefore, rather than de‐escalating therapy, the decision was made to further optimise treatment by up‐titrating selexipag to a higher tolerated dose.

Taken together, these findings were consistent with a possible mephedrone‐associated pulmonary arterial hypertension, after exclusion of other identifiable causes, though a causal relationship cannot be confirmed from a single case.

## 3 Discussion

This report describes the first case of PAH linked to chronic mephedrone use. Although mephedrone causes sympathetic activation, prior studies have not associated it with PAH or any other forms of pulmonary hypertension [1, 2, 3, 4, 5, 6, 7, 8, 9]. In contrast, related stimulants—including amphetamines, methamphetamine, and anorexigens—are established causes of drug‐induced PAH [10, 11] and are recognised guideline‐listed agents associated with progressive pulmonary vascular remodelling [12, 13].

There was marked initial hemodynamic and clinical improvement after cessation of mephedrone and initiation of dual oral PAH therapy. Importantly, however, her pulmonary hypertension did not fully resolve; she required ongoing treatment and later escalation to a prostacyclin receptor agonist to optimise her PAH risk status. Her trajectory therefore differs from fully reversible toxin‐associated PAH (e.g., dasatinib‐induced), yet also contrasts with methamphetamine‐associated PAH, which is typically progressive and often remains irreversible despite cessation [10]. Despite substantial initial clinical and haemodynamic improvement following mephedrone cessation and PAH therapy, persistent pulmonary vascular disease requiring treatment escalation suggests only partial reversibility, although conclusions from a single case remain limited.

The physiological basis for this patient's improvement is uncertain, and any mechanistic explanation remains speculative given the absence of prior data linking mephedrone to pulmonary vascular disease. Mephedrone produces sympathetic activation through monoamine release and α‐adrenergic stimulation [1, 2, 3], which could transiently raise pulmonary vascular resistance through functional vasoconstriction, although this has not been demonstrated experimentally in the pulmonary circulation. Synthetic cathinones can also induce oxidative stress and endothelial injury in systemic vessels [7, 8, 9], potentially promoting pulmonary vascular remodelling with chronic exposure. Stimulant‐related tachycardia, hypertension, and vasoconstriction may further increase right ventricular afterload. Cessation of these acute stimulant effects, together with PAH‐targeted therapy, may therefore explain the substantial initial haemodynamic improvement, while persistent disease may reflect underlying endothelial injury and established vascular remodelling.

Whether mephedrone has a direct and reversible effect on the pulmonary vasculature remains unknown, and further research is needed to determine if synthetic cathinones can influence pulmonary vascular tone or injury.

This case highlights the need to ask about recreational drug use in patients with unexplained pre‐capillary PH. Mephedrone is not a recognised cause, but its similarity to stimulants raises concern and may warrant further study as a potentially drug‐induced PAH.

## 4 Conclusion

In summary, this case raises the possibility of an association between chronic mephedrone use and pulmonary hypertension that warrants further investigation. Although not fully reversible, the patient demonstrated substantial clinical and hemodynamic improvement, contrasting with the typically progressive course of amphetamine‐ and methamphetamine‐associated PAH. As a single case report, it cannot establish causality; the temporal association observed does not exclude coincidental or unmeasured contributing factors. Heritable and major secondary causes of pulmonary hypertension were systematically excluded, though rarer aetiologies cannot be entirely ruled out, and we therefore describe this as a probable rather than confirmed association. Awareness of this potential link may support earlier recognition and ensure stimulant exposure is considered—and appropriately investigated for alternative causes—in patients presenting with unexplained pre‐capillary pulmonary hypertension, and further cases are needed to determine whether this represents a consistent pattern.

## Author Contributions

Masoud Kashoub: conceptualisation, data collection, drafting of the manuscript, literature review. Michael Plunkett, Heba Nashat, Konstantinos Dimopoulos, Colm McCabe, Carl Harries, S. John Wort, Paul I. Dargan, and Laura C. Price: clinical supervision, interpretation of clinical data, critical revisions of the manuscript. All authors reviewed and approved the final manuscript.

## Funding

The authors have nothing to report.

## Ethics Statement

According to institutional policy, ethical approval was not required for a single‐patient case report. All efforts were made to protect patient privacy.

## Conflicts of Interest

Paul I. Dargan serves as a Commissioner to the UK Commission on Human Medicines and is the Past President of the European Association of Poisons Centres and Clinical Toxicologists. He also acts as an advisor to the UK Advisory Council on the Misuse of Drugs, the European Union Drugs Agency, the United Nations Office on Drugs and Crime, and the World Health Organization. The other authors declare no conflicts of interest.

## Guarantor

Laura C Price is the guarantor of the work and accepts full responsibility for the integrity of the content.

## Data Availability

The data that support the findings of this study are available from the corresponding author upon reasonable request.
